# What acoustic telemetry can and cannot tell us about fish biology

**DOI:** 10.1111/jfb.15588

**Published:** 2023-12-17

**Authors:** David M. P. Jacoby, Adam T. Piper

**Affiliations:** ^1^ Lancaster Environment Centre Lancaster University Lancaster UK; ^2^ Institute of Zoology Zoological Society of London London UK

**Keywords:** biotelemetry, conservation, fish behavior, fisheries, movement ecology, tracking

## Abstract

Acoustic telemetry (AT) has become ubiquitous in aquatic monitoring and fish biology, conservation, and management. Since the early use of active ultrasonic tracking that required researchers to follow at a distance their species of interest, the field has diversified considerably, with exciting advances in both hydrophone and transmitter technology. Once a highly specialized methodology, however, AT is fast becoming a generalist tool for those wishing to study or conserve fishes, leading to diversifying application by non‐specialists. With this transition in mind, we evaluate exactly what AT has become useful for, discussing how the technological and analytical advances around AT can address important questions within fish biology. In doing so, we highlight the key ecological and applied research areas where AT continues to reveal crucial new insights and, in particular, when combined with complimentary research approaches. We provide a comprehensive breakdown of the state of the art for applications of AT, discussing the ongoing challenges, where its strengths lie, and how future developments may revolutionize fisheries management, behavioral ecology and species protection. Through selected papers we illustrate specific applications across the broad spectrum of fish biology. By bringing together the recent and future developments in this field under categories designed to broadly capture many aspects of fish biology, we hope to offer a useful guide for the non‐specialist practitioner as they attempt to navigate the dizzying array of considerations and ongoing developments within this diverse toolkit.

## 1 INTRODUCTION

Sound propagates four times faster, attenuates more slowly, and travels considerably further in water than it does in air. On this premise, acoustic telemetry (AT) technologies have, over the last 70 years or so, developed and diversified into a vast and lucrative industry enabling researchers to track numerous aquatic species over substantial spatial and temporal scales (Cooke, Hinch, et al., 2004; Hockersmith & Beeman, 2012; Hussey et al., 2015). Once a highly specialized methodology, typically adopted to understand the movement and space use of relatively large animals, it has since become embedded into a variety of ecological and applied research areas, co‐evolving alongside a suite of complimentary aquatic research approaches. Nowhere has this transition been more pronounced than within the fish biology community. AT has now become very much a generalist tool and one being adopted by an increasing diversity of practitioners from early career researchers to conservationists within the charity sector, to those managing recreational and commercial fisheries (Curtis et al., 2015; Hussey et al., 2017), aquaculture (Hassan et al., 2019), and commercial water facilities (Klimley et al., 2017). In light of this broadening market, and in the context of the rapid and ongoing technological developments within the AT field, there is a necessity to critically evaluate which aspects of fish biology this technology can now be useful in addressing.

In essence, animal borne, acoustic telemetry transmitters (hereafter “transmitters”) that generate coded acoustic signals at a specific frequency can be logged by a researcher directly with a hydrophone from a boat (active tracking) or by stationary in situ “receivers” with hydrophones attached, recording the presence of an individual within a particular and highly variable range. Since the early days of active continuous ultrasonic tracking in the 1950s, the field of AT has undergone a number of significant phase transitions; perhaps most significantly, the implementation of passive tracking using arrays of fixed receivers, which revolutionized the scope and scale of research question that could be tackled (Cote et al., 1998; Heupel & Hueter, 2001). This development put the onus firmly on study design, dramatically increasing the number of individuals that could contribute to a given study by reducing the effort required to collect data for each. Today, depending on the spatial arrangement and type of receivers installed within an array, data can take two forms: it can be returned either as presence only, recording the identification, time and date of a fish anywhere within an ellipsoid that represents the detection range of a particular receiver, or by closely spacing receivers to produce detection ranges that overlap, high‐resolution tracking can be conducted, generating submetre positional estimates of fish movement (Brownscombe, Lédée, et al., 2019). Nuances in the placement of acoustic receiver arrays are often dictated by the geography or environmental conditions of specific study sites. Arrays therefore can be highly variable, leading to placements of receiver gates within bottlenecks, grided arrays within enclosed lakes or embayments, or receiver “chains” that track the shape of a coastline, island, or river bed (Heupel et al., 2006). With recent advances in both transmitter and receiver technologies comes the opportunity to track fishes for longer, with higher precision or greater spatial coverage, follow them in deeper habitat or in near real‐time, while also gathering physiological data on the individuals that carry tags (Lennox et al., 2017). This increasing data richness and quantity per transmitter also provides the opportunity to address ethical considerations by reducing the number of individuals required to undergo procedures. Perhaps then, it is unsurprising that this toolkit has become more attractive in recent years to the diversity of people that work directly and indirectly with fish.

Whether using the simplest or the most advanced set up, the challenges and trade‐offs facing practitioners can be similar. These may include weighing up tag size against battery life (longevity) and the ethical implications associated with this (Brownscombe, Lédée, et al., 2019) or balancing acoustic coverage against research costs and questions (Heupel et al., 2006). Alternatively, it might be quantity versus quality of data and how best to analyze them (Guzzo et al., 2018; Whoriskey et al., 2019), the biases associated with the spatial configuration of an array (Kraus et al., 2018) or how detection range can vary through time impacting the accuracy and precision of the data, with significant implications for interpretation (Brownscombe, Griffin, et al., 2019; Kessel et al., 2014; Payne et al., 2010). These challenges (and more) have led to a wealth of developments in the visualization and statistical analyses of acoustic telemetry data (Campbell et al., 2012; Jacoby, Brooks, et al., 2012; Niella et al., 2020; Whoriskey et al., 2019) that continue to improve our understanding of fish biology across a diverse array of aquatic environments.

**FIGURE 1:**
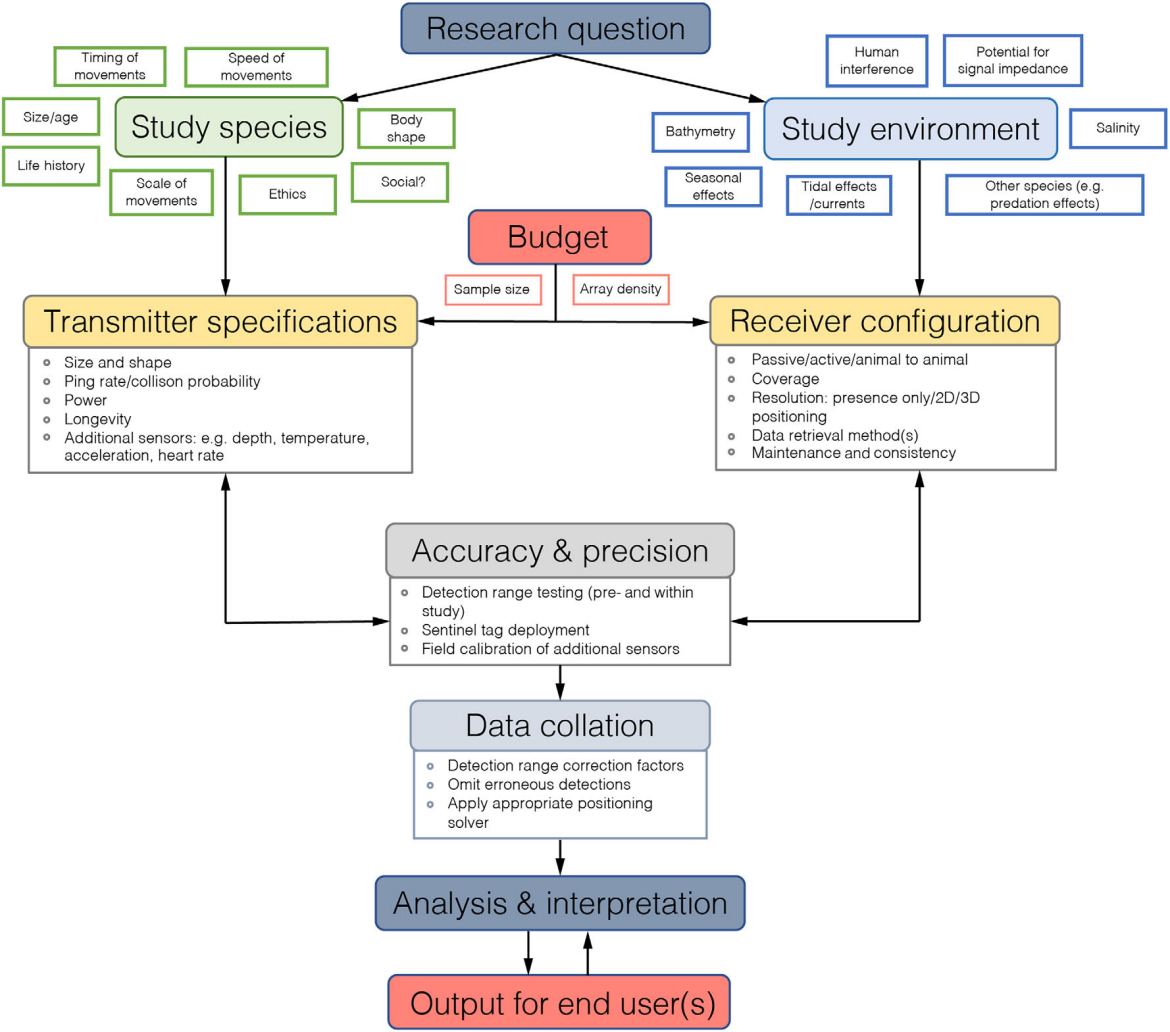
Plotting a course to accurately address a research question. Acoustic telemetry studies are typically highly nuanced and species‐ or site‐specific, which requires an ordered flow of decisions to help better link the research question with the end user and/or the practical application of the data for conservation and management of aquatic species. 2D, two‐dimensional; 3D, three‐dimensional.

Recent developments within AT offer new and more diverse opportunities to explore different aspects of fish biology. The increasing miniaturization, reduced cost, and improved battery life of current acoustic transmitters, for instance, has ensured that AT has become a vital part of the toolkit for those seeking to influence the conservation of imperiled aquatic species (Cooke, 2008) or inform management practices to mitigate pressures on their ecosystems (Matley et al., 2021). Alongside hardware developments, data management strategies, once rare and often unstandardized (Heupel et al., 2006), now offer broad scale, even global collaboration between researchers operating different project‐specific AT arrays (Abecasis et al., 2018; Cooke et al., 2011). Such innovation, however, is reliant on open methods and compatibility between manufacturer tag protocols (a set of unique tag IDs) and code maps (a list defining the particular tag IDs a receiver can listen for). Users new to AT therefore need to carefully consider the availability of sensor combinations and receiver‐transmitter, two‐way compatibility offered by the different AT manufacturers if collaborative research is desirable (Reubens et al., 2021). Where analyses used to be largely descriptive, they have started to become considerably more hypothesis‐driven and quantitative (Donaldson et al., 2014). Even the very description of the field now goes beyond referring simply to tags that transmit a unique ID code to passive monitoring stations to incorporate multifunctional temperature, pressure, acceleration, and even heart‐rate sensors (e.g., Kadar et al., 2019; Payne et al., 2015), with the option to retrieve real‐time updates on detections via satellite (e.g., Forget et al., 2015). For those relatively new to the field, this diversification and continuing development can offer up a daunting array of challenges and decisions (summarized in Figure 1), and as a growing number of excellent reviews will attest, the applications of these technologies are broad (Brownscombe, Lédée, et al., 2019; Donaldson et al., 2014; Heupel et al., 2006; Hussey et al., 2015; Matley et al., 2021; Matley et al., 2023).

In light of the transition of AT from a very specialized methodology to more of a generalist toolkit, our intention for this paper is to take stock of where the field is at in its capacity to reveal crucial information about fishes occupying an increasingly unpredictable and impacted world: our marine and freshwater ecosystems. As increasingly diverse practitioners enter the field, we wish to address the impact that AT can have on both fundamental ecological and applied research themes. We discuss these themes in turn, breaking them down into more specific areas, utilizing key papers that exemplify progress in each of these research areas (we also summarize this information in Table 1). At the same time, we aim to discuss some of the current limitations and future advances of AT, as well as celebrate the progress the field has made and continues to make within fish biology.

**TABLE 1:** A summary of the some of the strengths and weaknesses of acoustic telemetry for answering key fundamental and applied research questions in fish biology, along with suggestions for complementary approaches and analyses.

Fundamental ecological research	Strengths	Weaknesses	Complimentary approaches and suggested analyses
Migration patterns	Monitoring the spatial and temporal characteristics of migration Routes taken Individual variability and survivorship Passage rate at “bottlenecks”’ and obstructions (e.g., barriers, fish passes)	Sample size (particularly in partial migrating populations) Tag size and battery life skew species/life phase that can be tracked (although see *Future directions*) Limited ability to track in open ocean Lack of compatibility among collaborator arrays may limit scope and scale of research	Otolith microchemistry Population genetics Array collaboration to increase scope and geographical extent of study Time‐to‐event/survival analyses
Space use and fine‐scale movement strategies	Home range estimation (site attached species) Core area use Species/Sex/Individual or Site comparisons Connectivity Individual behavioral variation	Area use beyond the scale of array Highly mobile and far‐ranging species Autocorrelation can skew interpretation	Employ additional sensors in AT transmitters to understand drivers of movements Broad suite of density estimator approaches (e.g., KDE, AKDE, BBMM, dBBMM) Centres of activity
Habitat connectivity and energy landscapes	Potential for array to encompass entire spatial extent of individuals Conservative estimates of energy use (minimum linear distances, movement frequencies) Can inform nutrient dynamics	Trade‐off between spatial coverage and positioning accuracy Requires pre‐existing knowledge of spatial ecology to design an effective array	Spatial network analyses Stable isotope analyses Population genetics Bioenergetic models, speed‐distance calculations
Segregation	Detailed movements/space use on individuals from different components of a population	Transmitter life limits ability to explore changes in segregation patterns through lifecycle for long‐lived species	Employ additional sensors in AT transmitters to understand drivers of segregation Social network analyses/assortativity
Aggregation and social structure inference	Determine location, timing, and composition of aggregations Ease of deployment on existing aggregation structures, e.g., fish aggregating devices Monitoring reproduction events (spawning sites)	Without 2D or 3D positioning, difficult to differentiate scale of co‐occurrences Inference, not direct measurement Does not account for untagged individual influences on structure	Employ additional sensors in AT transmitters to understand drivers of aggregation Machine learning to define multi‐individual clustering events
Fine‐scale social associations and trophic interactions	High‐resolution tracking to determine fine‐scale associations Encounter rates Monitor direct interactions through transmitting and receiving transmitters (“business card tags”) Direct measurement of predation events	Lack of compatibility among collaborator arrays may limit scope and scale of research	Stable isotope analyses Physiological state determined through blood sampling or additional sensors Proximity‐based social networks Refinement and miniaturization of “business card tags”/mobile transceivers to directly measure associations
Depth preferences and temperature regulation	Hyperbolic positioning enables determination in *z* axis to track vertical movements Temperature and depth sensors coupled with AT transmitters widely available – provide 3D tracking in shallow water Behavioral thermoregulation	Dense receiver arrays required for 3D positioning not feasible in large water bodies and open ocean Additional calibration necessary in environments with extreme environmental variation	Measure concurrent environmental data to determine drivers of vertical movements Incorporation of additional sensors to decouple drivers of behavior
Invasion biology	Monitor and predict dispersal Interspecies interactions Habitat preferences Judas technique to locate aggregations of invasives and direct control measures	Limited capability to detect colonization front/early warning system Size/species limitations	Increasing scale of telemetry networks and collaboration provide great future potential

Applied research			
Evaluating extinction risk and threat assessments	Absolute population size and mortality assessment Trends in abundance Geographic range Aspects of spatial ecology that predispose species to decline and extinction Transnational receiver networks increasingly enable tracking of far‐ranging species	Limited tracking of deep‐water species due to technical and logistical challenges, e.g., array deployment and barotrauma from surface tagging Highly threatened species may be prohibited from tagging	Incorporate AT data into more traditional approaches, e.g., mark‐recapture for abundance assessment Use AT within multimethod approaches for threat assessment and conservation planning Spatially explicit integrated population models
Fisheries management	Use spatial data to accurately define stock units Quantify exploitation risk of different components of stock Survivorship and delayed mortality from by‐catch Spawning contribution of stock components “Real‐time” tracking enables adaptive management to reduce losses	Logistically complex to deliver “real‐time”/timely evidence for decision making Requirement for fish capture limits scale and reactiveness of approach Vulnerability of arrays to hostile parties	Additional sensors, e.g., heart rate and electromyograms, enable quantification of sublethal impacts from fishing, pollution etc. Integrate with genomics to understand selective pressure of fishing
Evaluating spatial protection	Space use within protected areas Aid conservation planning through identifying key habitats/locations Estimation of species‐specific vulnerability to threats	Tracking over several years may be necessary to effectively estimate activity space Knowledge of far‐ranging species limited by extent of receiver array	Integrate AT to ground truth environmental DNA detections of cryptic species Brownian bridge movement models Collaboration among array owners provides potential for transboundary assessment and protection
Human–wildlife conflict	Collecting information on spatial distribution and activity patterns to predict and manage encounters “Real‐time” tracking to inform public alert systems and avert conflicts Quantifying post‐encounter damage and mortality, e.g., in catch‐release fisheries, infrastructure development	Requirement for fish capture limits scale and reactiveness of approach Misuse of “real‐time” to target “problem” individuals	Combining live AT tracking with satellite/remote sensing approaches can aid rapid detection and active enforcement in conflict areas
Kinematics, energetics, and physiological impacts of human modified systems	Swim path metrics (e.g., path length, speed, turn angle, and direction of movement) derived from high‐resolution tracking	Accurate determination of energetics is reliant on concurrent monitoring of environmental factors and fish's physiological state at appropriate resolutions Often requires laboratory ground‐truthing of specific activities/behaviors observed with AT Pre‐impact comparative data often lacking	Movement models to predict dispersal Combine with additional sensors to quantify the energetics of movement behaviors Agent‐based models to predict behavior and therefore damage/mortality risk under different management scenarios Increasing miniaturization and incorporation of additional sensors and fish surrogates providing new insights

Abbreviations: 2D, two‐dimensional; 3D, three‐dimensional; AKDE, autocorrelated kernel density estimation; AT, acoustic telemetry; BBMM, brownian bridge movement models; dBBMM, dynamic brownian bridge movement models; KDE, kernel density estimation.

### 1.1 Fundamental ecological research

In this section we focus on areas where AT has revealed significant ecological insight within fish biology. The aim is to summarize the developments in several key fields using studies that exemplify notable progress in these particular research areas.

#### 1.1.1 Migration patterns

As a behavior, migration is both ecologically important, but also significantly threatened worldwide, yet understanding migration in fishes is often complicated by variation within species and between populations (Lennox et al., 2019). An appreciation of where, when, and what proportion of fish populations migrate is of critical importance for the management of threatened and/or commercially important fish stocks, the conservation of threatened species, and our fundamental understanding of species distributions. Deriving this information for many species is challenging, however, not least because fish movements do not abide by human‐imposed political boundaries and species rarely range in areas under a single jurisdiction. Furthermore, depending on the species, migration can occur across different orders of spatial magnitude from tens to thousands of kilometers (Chapman et al., 2012; Lédée et al., 2021; Lowerre‐Barbieri et al., 2021).

For fishes that migrate either entirely in freshwater (potadromy) or between freshwater and marine environments (diadromy), the use of AT has proven critical for revealing the scale and variability associated with migration, particularly in the freshwater component of this behavior. Strategic use of receiver “lines” or check points that span waterways and reliably capture both upstream and downstream movements of tagged individuals enable estimates of migration distance, timing, and relative survivorship (Clements et al., 2005; Melnychuk et al., 2007). Indeed, the mechanics of moving between salinity gradients for diadromous species have only really been fully understood by combining AT with otolith microchemistry. Telemetry‐defined migratory behavior, in combination with otolith analyses, has been used to validate or disregard chemical signatures associated with transitions in pinkeye mullet *Trachystoma petardi* (Castelnau 1875) (Miles et al., 2018), but also to determine partial anadromy in non‐native rainbow trout *Oncorhynchus mykiss* (Walbaum 1792) (Roloson et al., 2020).

These combined interdisciplinary approaches provide new levels of ecological understanding, particularly for complex migratory species, helping to better link the influence of flexibility in migration strategy to threats that may impact individuals/groups within populations disproportionately (Tamario et al., 2019). A closer look from a recent study, however, suggests that 50% of published articles that use AT to understand fish movement or ecology fail to incorporate or consider mortality within their study, while those that did estimate an ~11% loss on average of tagged individuals from the system (Klinard & Matley, 2020). This is pertinent as transmitters will continue to be detected even after depredation, leading to movement patterns that reflect the predator rather than the prey species (Bohaboy et al., 2020). Even those that survive but leave the array, and thus exhibit different behavior to individuals typically included in analyses, remain rarely discussed in studies on movement. Yet despite these important caveats, AT continues to prove invaluable for understanding fish migration. Hayden et al. (2014), for example, used receiver lines situated in the nearshore waters of Lake Huron and a multistate mark‐recapture model to describe three migratory pathways for walleye *Sander vitreus* (Mitchill 1818), demonstrating that males spent significantly longer in the rivers before migrating out into a bay than females, despite no sex preferences for specific pathways. Acoustic tracking of lake sturgeon *Acipenser fulvescens* (Rafineque 1817) in the same region (Huron‐Erie Corridor, HEC) has also proven instrumental in highlighting intraspecific variability in freshwater migrants, known as divergent migration (Kessel et al., 2018). As anthropogenic barriers continue to pose one of the biggest threats to riverine migration, the identification of consistent migratory behavioral states, including partial migration, where only some individuals from a population migrate, and non‐migratory residency within populations, illuminates the need for separate management strategies as well as the potential for species to respond to continued change to their habitat (Kessel et al., 2018).

**FIGURE 2:**
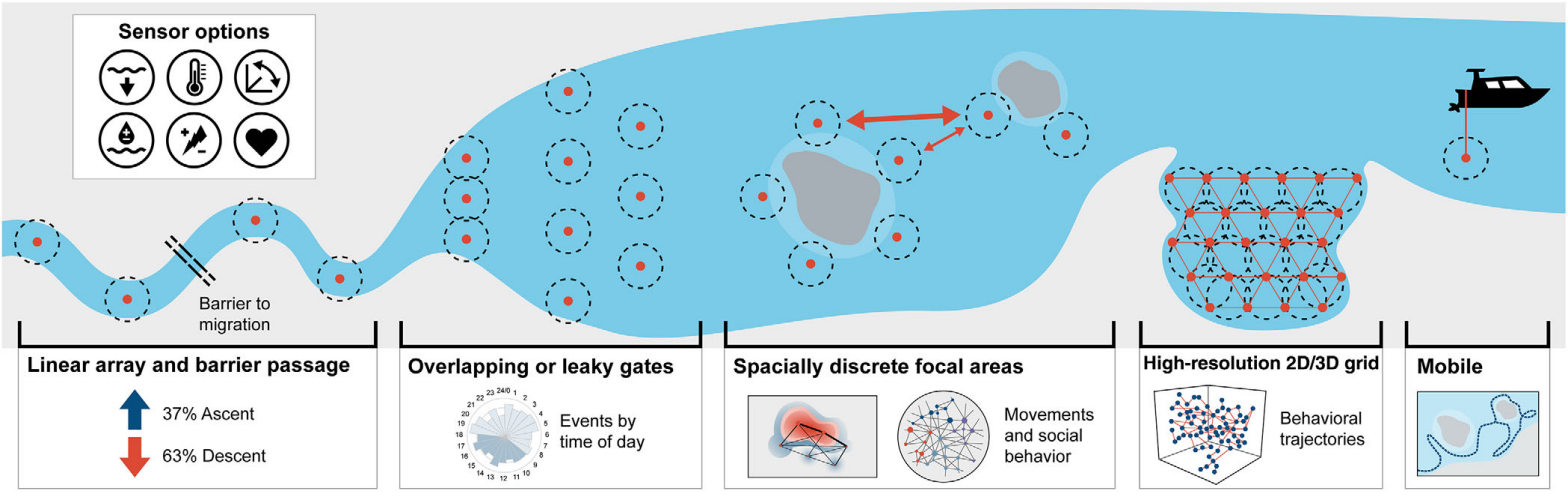
Variation in array configuration, complexity, and tag sensor “add‐ons.” Outside of active, boat‐based tracking, passive arrays can vary from linear, non‐overlapping arrays to gridded, overlapping, high‐resolution arrays, each shaped by infrastructure, cost, geography, species, and other logistical constraints. Furthermore, fish can be tagged with transmitters that offer additional functionality such as depth, temperature, activity, salinity, conductivity, or heart rate (left to right, top to bottom). Red dots indicate acoustic receivers and the dotted lines indicate estimated detection ranges. 2D, two‐dimensional; 3D, three‐dimensional.

As indicated, moving from a freshwater environment to marine imposes considerable physiological demands on fishes but also our ability to utilize AT to monitor migration, without the natural “bottleneck” that rivers provide. Array design between habitats can vary substantially (Figure 2), highlighting the need to carefully consider species ecology. For diadromous species like freshwater eels (*Anguilla* spp.) that mature in rivers and estuaries before undertaking their only spawning migration to the open ocean, understanding the timing, drivers, and threats to migration is vital for conserving these imperiled species (Jacoby et al., 2015). Béguer‐Pon et al. (2014) successfully deployed acoustic receivers covering a distance of 420 km to monitor the silver eel escapement of mature America eels *Anguilla rostrata* (Lesueur 1821) as they headed out towards the Sargasso Sea to spawn from the St. Lawrence River. The acoustic data revealed substantial individual variation in the timing and speed of migration, but for the first time a strong reliance on nocturnal ebb tide transport by silver eels to escape the estuary (Béguer‐Pon et al., 2014). When tracking species in the marine environment, horizontal migration is typically detected on departure and arrival by strategically deployed receiver arrays, as documented, for example, in bull sharks *Carcharhinus leucas* (Müller & Henle 1839) (Daly et al., 2014; Heupel et al., 2015). Alternatively, with depth‐temperature sensor tags, active acoustic tracking can provide a window into the short‐term vertical migrations (e.g., diel vertical migration) of highly mobile species of pelagic fishes (Block et al., 1992; Nakano et al., 2003). Finally, long‐distance movements in the marine environment, normally outside the capabilities of passive AT, are beginning to be captured via coordinated networks of acoustic arrays operating data‐sharing agreements to track cross‐jurisdictional migration of wide‐ranging, commercially important, or threatened species (Lédée et al., 2021; Young et al., 2020).

#### 1.1.2 Space use and fine‐scale movement strategies

Across most aquatic environments, AT has been used to great effect to estimate fish activity space, home range, core areas, or “central places” and residency patterns, in addition to how these parameters vary by species, sex, or time of day, month, or year (Garcia et al., 2015; Heupel et al., 2004; Heupel, Lédée, & Simpfendorfer, 2018; Kirby et al., 2017; Nakayama et al., 2018; Papastamatiou, Bodey, et al., 2018a; Papastamatiou, Watanabe, et al., 2018b; Simpfendorfer et al., 2002; Watson et al., 2019). The accuracy of space use estimates derived from passive telemetry data is very much dependent on the metric used (Dwyer et al., 2015). Some of the most widely used are now built into bespoke packages, such as those in the R statistical environment (R Core Team, 2022), for example *VTrack*, offering standardized tools for deriving and comparing these metrics between locations (Udyawer et al., 2018). It is important to stress, however, that there remain a number of challenges associated with estimating space use from AT data, not least that estimates are constrained by the size of the array, limiting reliability to species that use smaller areas than are being monitored. Accurate estimation of space use and home range of fishes is first contingent on precise estimation of location (Hostetter & Royle, 2020) and must consider biases that include autocorrelation, small numbers of tagged individuals (sample size), and irregular data collection. The pros and cons of home‐range estimator methods have been discussed in detail by Silva et al. (2022) and Kraft et al. (2023), offering accessible guides to choosing between the different options, in addition to R code for applying autocorrelated kernel density estimators for home‐range analyses. With these caveats in mind, and for species that show some form of site‐attachment or fidelity, AT has remained invaluable for understanding space use at multiple spatial scales, particularly in recent years with the advent of open source data platforms enabling the coordination of data streams from multiple acoustic arrays to cover significantly broader spatial ranges for more mobile species (Brownscombe, Lédée, et al., 2019; Campbell et al., 2012; Harcourt et al., 2019; Heupel, Kessel, et al., 2018; Udyawer et al., 2018).

Aggregated by species or sex, movement metrics (including range and dispersal) provide an important overview of space use at the population level. However, metrics from individual animals inform another important area of research; the role of individual variability or personalities (consistent individual behaviors) and behavioral syndromes (a correlated suite of behaviors) in population stability and adaptive resilience (Villegas‐Ríos et al., 2017). Using Atlantic cod *Gadus morhua* L. as a model species, Villegas‐Ríos et al. (2018) exposed individuals to repeated and standardized behavioral laboratory assays prior to releasing them with acoustic tags into a high‐resolution, acoustic tracking array (Innovasea Positioning System, formerly known as the Vemco Positioning System or VPS) to monitor their movements in response to changes in sea surface temperature. From hyperbolic positioning within the VPS array and depth‐sensing tags, fine‐scale reconstructions of three‐dimensional (3D) movements were modeled against individual home range across the proactive (bold) to reactive (shy) behavioral spectrum. In short, one of the key results to come from this novel work was that personality was found to be a significant predictor of changes in home‐range size (Villegas‐Ríos et al., 2018).

#### 1.1.3 Habitat connectivity and energy landscapes

The design of a passive acoustic array and the equipment used can vary significantly (Figure 2). As such, data can be generated as discrete, presence‐only packages associated with important monitoring locations or as discussed, near‐continuous, high‐resolution 3D individual tracks reliant on receiver overlap and considerable post‐processing of the data to determine fine‐scale position. Particularly when tracking species in the marine environment or in very large water bodies, positional accuracy is regularly sacrificed for spatial coverage. Arrays can be designed around habitats of interest such as reefs, islands, or atolls (Espinoza, Heupel, et al., 2015; Papastamatiou et al., 2015), or as gridded arrays and receiver lines, which are sometimes adopted where the physical geography of the study location and the research question permits, such as bottlenecks or enclosed embayments (Block et al., 2019; Braccini et al., 2017; Farmer & Ault, 2011; Hussey et al., 2017) (Figure 2).

When covering broad geographic areas or different habitat types, discrete spatial data lend themselves well to spatial network analyses of movements between receiver locations (Jacoby, Croft, & Sims, 2012). The true strength of network analyses is that they offer a scalable method with which to quantify linkages, measure relative centrality or importance of receivers, explore connectivity, and determine the extent to which landscape (structural) and behavior (functional) processes facilitate or impede movement between habitat patches or resources (Baguette & Van Dyck, 2007; Bélisle, 2005). Indeed coupling movement networks with stable isotope analyses has led to important and novel discoveries around energy landscapes, for example the classification of permit *Trachinotus falcatus* into two distinct ecotypes within the Florida Keys, United State: one, with a heavy reliance on movements between the Florida reef tract and seagrass beds and their associated prey, and a second that primarily occupies artificial reefs relying almost exclusively on pelagic prey, with clear implications for the management of the fishery (Brownscombe et al., 2022). Consequently, it is becoming increasingly apparent that AT‐derived fish movements, in combination with bioenergetic models, can greatly inform our understanding of nutrient dynamics, with network approaches being adopted to predict the distribution and quantities of nitrogen egestion by predators on coral reefs (Williams et al., 2018). Using a similar coupled approach, Eggenberger et al. (2019) were able to demonstrate variation in the behavior and habitat selection of the common snook *Centropomus undecimalis* (Bloch 1792), despite similar trophic ecology, in response to mesotrophic (higher mobility) and eutrophic (higher residency) conditions.

The application of network analyses to tease apart some of these processes is still in its relative infancy, particularly the utilization of edge durations (time associated with movements from one receiver to another) to explore some of the mechanisms driving connectivity. These detection “gaps” have proven useful for inferring different fish behaviors associated with “restricted” movements and “out‐of‐range” dispersal (Williamson et al., 2021). To date, network approaches have been successfully applied to AT data to show how reef‐associated shark species connect different management zones in the Great Barrier Reef (Espinoza, Lédée, et al., 2015), and how movement strategies can influence species risk to illegal fishing inside marine protected areas (Jacoby et al., 2020). Furthermore, network metrics, which capture dynamic movements, appear both consistent with and complementary to more traditional estimates of space use (Lédée et al., 2015), offering an extended toolkit to the AT practitioner (Jacoby & Freeman, 2016). For example, the repeated path use of young *G. morhua* between habitats within a coastal fjord system was strongly negatively correlated with water temperature, a finding revealed through measuring the relative abundance of different types of triadic network motif or three receivers linked by directed movements (Staveley et al., 2019).

#### 1.1.4 Segregation

In addition to using AT to quantify space use, we might wish to explore some of the mechanisms driving this space use. Individual behavioral signatures, whether in two or three dimensions, may be dictated by their local environment or by the presence of conspecifics of a different sex or size or individuals of different species altogether, manifesting itself as spatial and/or temporal differences in habitat use. Realistically, it is likely to be a combination of factors, yet understanding the dynamics of segregation within a population is important, particularly when considering species that face spatially or seasonally focused exploitation or partial spatial protection (Mucientes et al., 2009). Using Innovasea's (Amirix Systems) accelerometer and pressure transmitters (V9AP and V13AP), for example, Payne et al. (2015) were able to demonstrate diurnal segregation on a vertical plane between an estuarine piscivore, mulloway *Argyrosomus japonicus* (Temminck & Schlegel 1844), and a benthic carnivore, sand whiting *Sillago ciliate* (Cuvier 1829), in south‐eastern Australia. Interestingly, the authors use these multipurpose tags to monitor the impact of short‐term stochastic weather events on segregation; the study reveals that rain precedes a switching of spatial segregation to temporal segregation (increased nocturnal activity in *A. japonicus* and decreased nocturnal activity in *S. ciliata*), a result compellingly supported by 10 years of commercial set‐net Catch Per Unit Effort (CPUE) data, which show increased rainfall produces higher catch rates for *A. japonicus* but lower catch rates for *S. ciliata* (Payne et al., 2015).

Sexual segregation is relatively well documented in marine fishes (Wearmouth & Sims, 2008) and here too AT has played a key role in distinguishing both sexual segregation within adult populations of elasmobranchs (e.g., Kock et al., 2013) as well as female‐only refuging behavior as a reproductive strategy for numerous species (e.g., Hight & Lowe, 2007; Sims et al., 2001). Furthermore, mobile, predatory elasmobranchs also have a tendency to demonstrate segregation by species; processes such as competitive exclusion within specific habitat types (Papastamatiou, Bodey, et al., 2018a) or dynamic, temporal segregation driven by tidal cycles (Lea et al., 2020) have been demonstrated in remarkably small systems – relative to the movement capabilities of the study species – such as remote isolated atolls, using long‐term AT data (e.g., Heupel, Kessel, et al., 2018). Despite having similar isotopic niches, AT has also revealed that the leopard coral grouper *Plectropomus leopardus* (Lacépède 1802) and the spotted coral grouper *Plectropomus maculatus* (Bloch 1790) had minimal spatial overlap, yet similar space use patterns, due to vertical segregation in the water column (Matley et al., 2017). Again, network analyses have been put to good use to show, for example, that even among apparently sympatric species, sharks vary considerably in their choice of habitat, route choice, and connectivity within a gridded receiver array in the southern Great Barrier Reef, Australia (Heupel, Lédée, & Simpfendorfer, 2018). Other applications include the use of community detection algorithms to networks of movements between different species and age classes to explore dissimilarity in movement within complex fish assemblages (e.g., Casselberry et al., 2020).

#### 1.1.5 Fish interactions

##### Aggregation and social structure inference

With enough individuals tagged simultaneously within a population, AT can be hugely informative for identifying and exploring fish aggregations and their key drivers, most notably spawning (Domeier & Colin, 1997), predation (Temming et al., 2007), refuging, and nursery behaviors (Bass et al., 2017; Jacoby, Croft, & Sims, 2012). In teleost reef predators such as grouper, determining the location, timing, and composition of reproductive aggregations is crucial to not only answer fundamental questions about population biology, but also inform spatial protection measures because aggregations are commonly targeted by fishers (Keller et al., 2020; Rowell et al., 2015). Indeed, the tendency of numerous pelagic species, including tropical tuna, to aggregate around floating objects has long been exploited to aid harvest through the deployment of artificial fish aggregating devices (FADs). The relative ease of instrumenting FADs with acoustic receivers and other sensors has enabled substantial knowledge gains about movement ecology (Pérez et al., 2020), the social interactions of individuals (Stehfest et al., 2013), and the vulnerability of target and bycatch species to exploitation (Forget et al., 2015). In freshwater, the locations of adult lake trout *Salvelinus namaycush* (Walbaum 1792) aggregations in Lake Huron, North America, determined from 5 years of acoustic positioning data within an extensive (19–27 km^2^) receiver array, revealed hitherto unknown putative spawning sites that were subsequently confirmed by diver surveys of egg deposition (Binder et al., 2018). Several of these sites were too small or obscure to have been identified by bathymetric survey or did not conform to the conceptual model of a spawning habitat so without telemetry would have otherwise likely been overlooked (Binder et al., 2018). Indeed, temperature and depth sensors on acoustic transmitters can reveal the abiotic conditions that favor aggregation. For example, having gained this information through AT, Bajer et al. (2011) used the Judas technique, that is, tracking an individual to reveal the location of an aggregation, to assist in the removal of invasive common carp *Cyprinus carpio* L. aggregations with an efficiency of up to 94%.

Determining the mechanism driving aggregation or social behavior from remote, passive data is in some instances non‐trivial and in others near impossible depending on the ecology of the species. Consequently, a new line of questioning has emerged that uses machine learning inference to define multi‐individual clustering events in acoustic time‐series data that indicate the spatial and temporal co‐occurrence of individuals (Jacoby et al., 2016; Mourier et al., 2018). Extracting these events using Bayesian inference reduces the subjectivity around predefining a sampling window with which to measure “social” behavior (10 mins? 10 h?), relying more on the natural and variable clustering of the visitation patterns produced by gregarious fishes. Co‐occurrence networks can then be generated from the clusters and worked up using common quantitative network analysis methods (Jacoby & Freeman, 2016), but careful interpretation of the social networks produced using these methods is needed as the distance over which individuals may be socializing (i.e., co‐occurring) is not always known (Mourier et al., 2017; see *Fine scale social associations* for more discussion around this). Caveats aside, this method has enabled exploration of the mechanisms behind social behavior in highly mobile, free‐ranging fishes for the first time, revealing, for example, stable social bonds in reef sharks that can last for years and likely function to facilitate information exchange (Papastamatiou et al., 2020).

##### Fine‐scale social associations and trophic interactions

**FIGURE 3:**
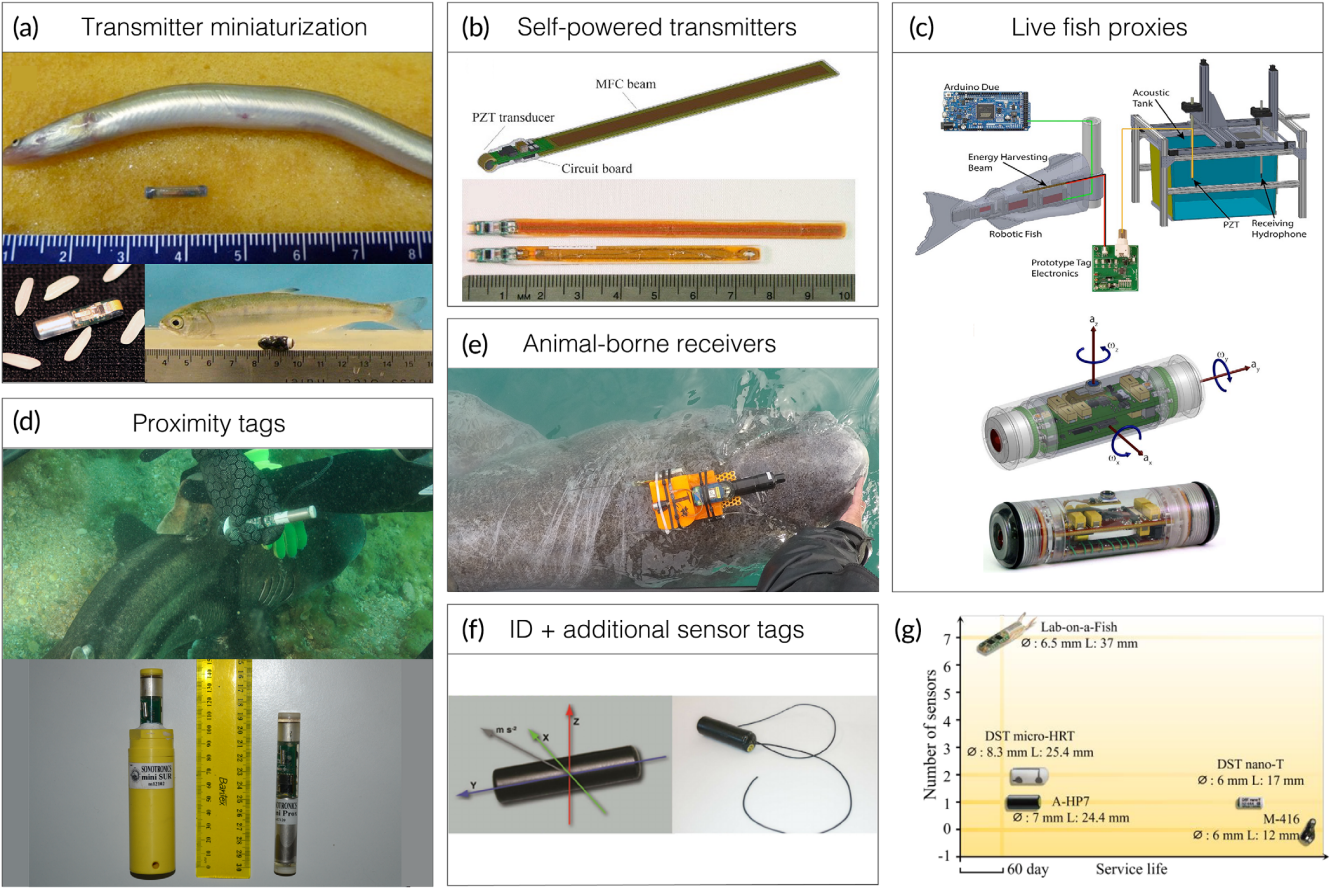
Important areas of transmitter development. (a) Micro acoustic transmitter (0.08 g) developed for small/juvenile eel and lamprey research (top), needle injectable acoustic transmitter (0.22 g) (lower left), and juvenile Sockeye salmon with 0.7 g micro acoustic tag (lower right). (b) Long‐life, self‐powered acoustic transmitter employs a flexible piezoelectric beam to harvest energy from fish swimming to power direct acoustic transmission or re‐charge onboard batteries. (c) Robotic fish tail developed to replace the need for live fish testing in early phase tag development (top) and Sensor Fish, a live fish surrogate that uses multiple sensors to approximate salmon smolt experience (e.g., shear forces, collisions with structures, acceleration, and pressure) when transiting deleterious route such as turbines, spillways, and sluiceways (bottom). (d and e) Proximity loggers and animal‐borne receivers record other fish in close proximity to one another (at a scale of meters). These have been trialed on Port Jackson sharks (d, top) to understand social networks and aggregation behavior (images: Justin Gill, Nathan Bass) and on Greenland sharks (e) as part of a telemetry package to explore interactions, behavior, and encounter rates between individual sharks (image: Nigel Hussey). (f) Acoustic transmitters now offer additional sensors, including, for example, acceleration and heart rate monitors. (g) Indication of how state‐of‐the‐art Lab‐on‐a‐Fish technologies fair against other commercial miniturised biotelemetric devices (reproduced with permission from Yang et al., 2021). All images unless otherwise stated kindly provided by Daniel Deng.

The fine‐scale co‐occurrences of individuals, whether between conspecifics as mutually beneficial social affiliations or between predator and prey species as direct interactions and displacements, are an important factor that can strongly influence population dynamics and/or spatial distributions of species (Morueta‐Holme et al., 2016). The encounter rates of Atlantic tarpon *Megalops atlanticus* (Valenciennes 1847) with predatory *C. leucas* and great hammerhead *Sphyrna mokarran* (Rüppell 1837) sharks in the Florida Keys, for instance, were elevated at specific locations and prior to spawning aggregation behavior, a result identified using machine learning to quantify spatio‐temporal overlap in multispecies AT tracking data (Griffin et al., 2022). To truly understand fine‐scale interactions and associations, however, requires direct measurement rather than inference methods, and at a precise and known spatial scale (Aspillaga et al., 2021; Mourier et al., 2017). Prototype methodologies and proof‐of‐concept studies have made exciting initial progress towards this endeavor. For example, recently developed transmitters that switch transmission code when digested in the stomach of a predator remove much of the uncertainty around formerly inferring predation events from changes in track characteristics (e.g., Romine et al., 2014), enabling more robust and detailed exploration of fishes' behaviors immediately prior to predation (Weinz et al., 2020). To reveal social behavior using AT, a degree of control is needed over the system. Using model systems of fish constrained to localized areas or relatively small lakes, high‐resolution tracking in combination with proximity‐based social networks (temporal network analysis), significant strides have been taken towards measuring the wild social behavior of fish. Vanovac et al. (2021), for example, tracked 108 freshwater fish (four species) every few seconds for a year to measure the location and duration of intra‐ and interspecific sociality. To measure social behavior in wider ranging species, beyond the practical limits of predefined static receiver arrays, prototype “business card tags” have been developed; these operate as both transmitters and receivers for mobile peer‐to‐peer communication (Holland et al., 2010). Furthermore, proximity transmitters, miniaturized receivers that can detect conspecific coded transmitters over distances <10 m (Guttridge et al., 2010) (Figure 3, specifically d,e), have shown that an individual's actual social encounters can be logged and stored pending transmitter retrieval. The need for further technological developments in this area remains, however; applications of devices like the Innovasea Mobile Transceiver (VMT) and Sonotronics' miniSUR, which are hybrid devices that transmit coded signals like acoustic transmitters, but also record transmissions from other tagged animals on the same frequency like monitoring receivers, are currently limited to small numbers on relatively large animals (e.g., Barkley et al., 2020; Haulsee et al., 2016) and in situations where the unit can be recovered to obtain the data. In all likelihood, advances in the 3D accuracy of spatial positioning of multiple tagged fish will yield the most insight into fine‐scale social behaviors over the next few years (Aspillaga et al., 2021).

As with many aquatic tracking technologies, data retrieval continues to be a significant hurdle to overcome, particularly for studies involving multiple individuals and their interactions, as the data can grow exponentially with the addition of every individual. That said, current off‐the‐shelf mobile receivers, in combination with other sensors, have provided tantalizing insight into the interactions of particularly elusive and cryptic species. Barkley et al. (2020), for example, use VMTs, accelerometers and radion antennae, combined in a pop‐off package to describe increased activity (acceleration and depth changes) in slow growing, seemingly solitary Greenland sharks *Somniosus microcephalus* (Bloch & Schneider 1801) when in the presence of conspecifics. Furthermore, the encounter rates of commercially important fish species (*G. morhua*, *Salmo salar*, and *A. rostrata*) and opportunistic mammalian predators have been gleaned through standard tagging (of fishes) with coded transmitters and the deployment of VMT receivers and GPS tags to gray seals *Halichoerus grypus* (Fabricius 1791) in Canada (Lidgard et al., 2014). Finally, as we have already discussed, AT combined with investigations into stable isotope ratios, blood plasma, and other physiological processes has greatly furthered our understanding of trophic dynamics, food web structure, and niche partitioning within species that share habitat (Dwyer et al., 2020; Matich & Heithaus, 2014). With the advent of increasingly open‐source tracking technologies, we envisage exciting progress in this area in the next 10 years.

#### 1.1.6 Depth preferences and temperature regulation

Detailed knowledge of how fish move through all three dimensions of the space they inhabit is often pivotal to our understanding of the mechanisms underpinning their behavior. Furthermore, the predominance of ectothermy among fishes means depth selection and thermoregulation are closely coupled. Water temperature together with dissolved oxygen levels, light, salinity gradients, prey availability, predation risk, and physical habitat features are among the key factors shown to drive vertical movements (Hussey et al., 2015) ranging from localized diel migrations, for example, in Myliobatid rays (Matern et al., 2000) to large‐scale seasonal habitat shifts in *S. vitreus* (e.g., Raby et al., 2018). As we have seen, ongoing refinement of hardware and analytical techniques can enable submetre positions on the *z* axis to be determined directly from the acoustic ping, and in near real‐time, using hyperbolic positioning. This has been used to good effect to elucidate how different structures, flow field, and temperature characteristics around hydropower facilities affect the vertical distribution and corresponding downstream passage outcome for migrating juvenile salmonids (Arenas et al., 2015; Deng et al., 2011; Li et al., 2015; Ransom et al., 2007). However, it is worth highlighting here that different manufacturers use different transmitter coding systems in an attempt to minimize both tag clashes and false‐positive detections, and this can impede compatibility and collaboration between networks of researchers using different technologies (see Reubens et al., 2021 for discussion around this issue). Furthermore, the comprehensive receiver arrays required for continuous 3D positioning often render its application unfeasible in the open ocean and large, deep lakes where species can be far‐ranging in all dimensions, while in shallow water there may be too little vertical separation in the locations of the hydrophones to adequately resolve transmitter depth (Cooke et al., 2005; Semmens, 2008).

Combining pressure and temperature sensors with acoustic transmitters offers a widely applicable and often more cost‐effective alternative (in terms of both hardware and data processing requirements), and can still provide high accuracy and precision (Baktoft et al., 2015). For example, Schurmann et al. (1998) were able to demonstrate that a change in the amplitude of diurnal migrations of sea bass *Dicentrarchus labrax* L. resulted from manipulating vertical oxygen gradients in the water column within an experimental tank, down to an accuracy of ±5 cm using acoustic pressure sensor transmitters. However, in field environments with extreme variation in environmental parameters (e.g., salinity, water temperature, flow rate) high accuracy in depth measurements may require additional field calibration (Brownscombe, Lédée, et al., 2019; Veilleux et al., 2016). Technical issues aside, acoustically transmitted temperature and/or depth sensor data have been used to investigate the influence of feeding regimes on the vertical activity of cage‐cultured *S. salar* (Føre et al., 2017), vertical thermoregulation in sunfish *Mola mola* L. (Cartamil & Lowe, 2004), vertical separation of year classes through predator–prey dynamics in bull trout *Salvelinus confluentus* (Suckley 1859) (Gutowsky et al., 2013), the impact of seismic surveying on *G. morhua* and saithe *Pollachius virens* L. distribution (Davidsen et al., 2019), and sea trout *Salmo trutta* L. use of vertical gradients as a response to parasite loading (Mohn et al., 2020). Direct measurement of the temperatures and depths that free‐ranging fish move through has allowed us to move beyond broad correlational inferences derived from 2D location data alone and advance understanding of fundamental aspects of fish physiology and environment selection. Nevertheless, there is the risk that without corresponding environmental data collected at biologically relevant temporal and spatial resolution, studies will lack the ability to fully contextualize such animal‐borne data. For example, despite gaining detailed movement data, including depth, from Mekong giant catfish *Pangasianodon gigas* (Chevey 1931) tracked for up to 9 months in a reservoir, insufficient collection of concurrent temperature and dissolved oxygen datasets meant it was not possible to draw robust conclusions about the mechanisms driving their behavior (Mitamura et al., 2008). In the future, there is great potential for repeating tracking studies that have produced well‐defined relationships between fish distribution, behavior, and water temperature as a tool to identify and predict the impacts of a changing climate.

#### 1.1.7 Invasion biology

An important prerequisite to applied measures for combating the growing list of fish species becoming established in non‐native locations is to understand the impact they have on native species and habitats. This might include monitoring the spread, movement capabilities, reproductive ecology, and competitive interactions with other species (Deacon et al., 2011; Mills et al., 2004). AT has been pivotal in revealing some of this ecological information, which can then inform more targeted mitigation measures. One of the first fish to ever be domesticated, the goldfish *Carassius auratus* L., now considered as one of the world's most invasive species, were tracked in a river in south‐western Australia using AT to show that some individuals were capable of moving >200 km per year; crucially this study was also able to infer that movements into lentic habitat coincide with spawning behavior in this species providing vital knowledge for control programmes (Beatty et al., 2017). Monitoring a newly established source population of round goby *Neogobius melanosto‐ mus* (Pallas 1814) within the Rideau Canal in Ontario, Canada, Bergman et al. (2022) were able to track the invasion front of this species, which is normally native to the Black and Caspian Seas. Dispersal among a quarter of the tagged individuals was established via receivers situated within canal locks, which were hypothesized to enhance passage (Bergman et al., 2022). The scale of the challenge facing marine invasive control has been demonstrated through a study on lionfish *Pterois volitans* L. in the western Atlantic, showing an eight‐fold variation in individual home range estimates (~48,000–379,000 m^2^) and ~40% of individuals traveling >1 km from the tagging site towards deeper habitat (Green et al., 2021). With the success of species invasion often contingent on species‐community interactions (Lodge, 1993), multispecies AT tagging programmes will be key, as will developments to overcome the challenges discussed in the previous section around measuring fine‐scale interactions.

### 1.2 Applied research

There are many cases in which the ecological information gleaned from AT studies on fish are an important precursor to applied management measures, mitigation strategies, or conservation interventions. In this section we explore more explicitly how AT has fundamental application in the management and conservation of aquatic resources.

#### 1.2.1 Species conservation and management

##### Evaluating extinction risk and threat assessments

Continuing data deficiency in even basic population parameters hinders the robust classification of extinction risk for a fifth of global fish species as assessed by the IUCN (2020) and prevents the potential for their protection within legal frameworks (VanderZwaag et al., 2013). The assessment of endangerment relies on fundamental knowledge of demographic parameters to estimate absolute population size, trends in abundance, and geographic range (IUCN, 2020). By tracking individuals from different components of the population, for extended periods of time and with the ability to determine much more precisely when mortality occurs compared to traditional mark‐recapture approaches, AT provides a powerful means of collecting such data for fishes (Lees et al., 2021). Furthermore, telemetry‐derived data can facilitate quantification of the main processes driving species decline and extinction (habitat loss and alteration, overexploitation, introduced species, pollution, and climate change), most obviously in the context of how the spatial ecology of a species predisposes it to specific impacts (Cooke, 2008). In a notably rare example of deep water AT, southern dogfish *Centrophorus zeehaanii* (White et al., 2017) were tracked for 15 months at depths of between 300 and 700 m to demonstrate the effectiveness of a large (100 km long) fishery closure to conserve this species, extirpated from much of its range off southern Australia (Daley et al., 2015). Although clearly possible, there remain substantial limitations to tracking wide‐ranging species and/or those that occupy deep water habitats. Technical and logistical challenges in deploying deep‐water arrays have constrained the majority of AT studies to depths under 50 m (Loher et al., 2017), and bringing physoclistous species to the surface to tag poses the risk of damage and mortality due to barotrauma and post‐release predation (e.g., Bohaboy et al., 2020; Curtis et al., 2015). The increasing use of in situ tagging methods at depth and improvements to surface tagging protocols such as employing descender devices and rapid tag attachment methods to minimize time at the surface will further unlock the huge potential of AT to study fish movements and population dynamics in the deep sea (Edwards, Hiltz, et al., 2019; Runde & Buckel, 2018).

Threats to fishes, especially those with complex lifecycles that undertake migrations between habitats, vary through their lifetimes, making the study of all life stages imperative. Minimum acoustic transmitter size has historically prohibited the study of small, juvenile life stages (see *Tracking small species and life stages*), the population component which for many endangered fish species suffers high human‐induced mortality (e.g., Chinook salmon *Oncorhynchus tshawytscha* (Walbaum 1792) Perry et al., 2010). Furthermore, for long‐lived species transmitter life duration may be prohibitively short (Donaldson et al., 2014). Technological advances, the growth of large transnational receiver networks (e.g., the Great Lakes Acoustic Telemetry Observation System [GLATOS], the Ocean Tracking Network [OTN], and the European Tracking Network [ETN]) and new approaches to data analysis such as incorporating acoustic data into mark‐recapture models (Bird et al., 2014; Dudgeon et al., 2015), as well as the growth of spatially explicit integrated population models (Goethel et al., 2021) that better estimate abundance and predict the impacts of environmental change, are all expanding the utility of AT for threat assessments and conservation planning. However, AT remains just one in a suite of necessary tools, as exemplified by studies on *S. microcephalus*, a species for which significant knowledge gaps remain. Effective management is most likely to be realized through a multimethod approach integrating biologged physiological, environmental, and movement data with population genetics and genomics, stable isotope analysis, and commercial catch data (Edwards, Pratt, et al., 2019).

##### Fisheries management

AT has enabled vast knowledge gains about the spatial ecology of fishes, which in the context of exploited species, especially those that are wide‐ranging and/or straddle national boundaries, is fundamental to effective fisheries management. In the first instance, AT can be far more effectively employed to define the stock unit than traditional approaches such as mark‐recapture (Donaldson et al., 2014). For example, acoustic tracking of Greenland halibut *Reinhardtius hippoglossoide* (Walbaum 1792) revealed connectivity between its use of inshore fjords and offshore habitats around Baffin Island, Canada, casting doubt on the status of separate inshore “resident” and offshore stocks, and highlighting the need for a shared quota (Barkley et al., 2018). Conversely, the discovery of high site fidelity and presumed natal homing has challenged the assumption of common stocks in many species, including *G. morhua* (Robichaud & Rose, 2001; Svedäng et al., 2007), Pacific cod *Gadus microcephalus* (Tilesius 1810) (Cunningham et al., 2009), and *C. undecimalis* (Young et al., 2014). There is also growing recognition of how individual and ontogenetic variation in spatial responses to environmental conditions and exploitation drives the dynamics of populations (Alós et al., 2019; Goethel et al., 2021). In addition to this increasingly fine‐scale understanding of the structure and spatial dynamics of exploited stocks, many of the life‐history parameters required for stock assessment models can be directly determined using AT (Crossin et al., 2017). These include instantaneous mortality rate (Block et al., 2019), survival probabilities related to life‐stage and migration pattern (Chaput et al., 2019; Perry et al., 2010), delayed mortality from by‐catch or recreational catch and release activities (Curtis et al., 2015; Halttunen et al., 2010; Yergey et al., 2012), predation (Berejikian et al., 2016), and the spawning contribution of different stock components (Faust et al., 2019). Crucially for fisheries management, this information is attainable at the scale of the specific stock (DeCelles & Zemeckis, 2014). By bringing together datasets on spatial dynamics with these population parameters, spatially explicit integrated population models offer great potential to more accurately predict species' responses to dynamic processes such as harvest mortality and climate‐induced changes (Goethel et al., 2021). Nonetheless, despite the versatility and breadth of AT for informing fisheries management, in a review of global AT studies on all aquatic animals, Matley et al. (2021) found a lack of management driven applications, with most studies focussed on generating general movement data. They also highlight key challenges to be addressed, such as developing analytical tools and standardized approaches among research groups to allow the potential of the vast quantities of AT data being collected globally to be fully realized (Matley et al., 2023, 2021).

It is the integration of AT with other approaches and the development of real‐time tracking that offer most promise for more nuanced, creative, and adaptive management of fisheries into the future. The increasing use of additional sensors such as heart‐rate monitors and electromyograms enables quantification of the sublethal fitness impacts of fishing activities such as stress‐induced physiological changes from catch and release (Donaldson et al., 2008 and references therein). Within the context of ecotoxicological studies that have the dual purpose of understanding the impact of pollution on exploited stocks, as well as the human health risks of consumption, AT provides the opportunity to relate individual fish movements to contaminant burden and thereby manage exposure risk (Taylor et al., 2018). Crucially, AT enables an understanding of trait variation (e.g., movement) between individuals, relative to the population mean, which for fisheries that can unknowingly selectively harvest can have important implications for ecosystem functioning when combined with physiological data (Allgeier et al., 2020). Furthermore, behavioral change in response to hyperdepletion effects, such as reduced vulnerability or increased timidity, can also be measured with AT, providing critical information for stock assessments and harvest control (Arlinghaus et al., 2017). Equally, integration with genomics promises insight into how genetic variation drives individual behavior, with applications ranging from predicting the ways in which environmental change may impact highly locally adapted yet exploited species such as Arctic char *Salvelinus alpinus* L. (Moore et al., 2017), to understanding the extent to which fishing exerts a selective pressure on wild populations (Olsen et al., 2012; Villegas‐Ríos et al., 2017). Gaining increasingly detailed information on threats enables continued refinement of conservation and fisheries management policies. For example, Forget et al. (2015) used AT to determine the vulnerability of target and non‐target species to FADs used in the tuna purse seine fishery, identifying how impacts on non‐target species could be reduced. Finally, by removing the time lapse associated with periodic receiver download, real‐time tracking opens up huge possibilities for adaptive management, an approach that has also garnered much attention in aquaculture (Føre et al., 2017; Hassan et al., 2019). In one of the first examples from a wild fishery, on the Sacramento River, United States, receivers transmitting near real‐time data to a communications centre alerted water managers to the earlier than expected migration of *O. tshawytscha* smolts. In response, water diversion structures into the delta were closed, greatly reducing the loss of fish through that route (Klimley et al., 2017).

##### Evaluating spatial protection

Integrated data and the organized collaboration of “individual” acoustic telemetry projects (Taylor et al., 2017) is already proving invaluable for managers to assess connectivity created by long‐range movements between areas of concern (Lédée et al., 2021). This can also provide important information guiding the restoration of critical habitat (Brooks et al., 2017) and enable adaptive management of river water control structures to enhance connectivity during key fish migration events (Klimley et al., 2017; Teichert et al., 2020). Consequently, through either manual tracking or passive arrays, AT remains one of the primary tools for assessing the space use of imperiled species residing within existing or proposed aquatic protected areas (Cooke et al., 2005). Novel approaches, for example those that combine AT with resource selection functions that integrate movement data with data on resource availability, are beginning to be adopted to assist with the initial prioritization and evaluation of habitat to be conserved (Griffin et al., 2021). Additionally, diversification of environmental DNA (eDNA) approaches to assess the spatio‐temporal distribution of cryptic species will likely require the increasing support of AT to assist in validating positive eDNA detections (Harris et al., 2022) as this relatively recent methodology continues to be developed and refined.

The ability to accurately assess the efficacy of protected areas using AT, however, is highly dependent on the size of the area under protection and the ability of the species in question to make long‐range movements. Even for highly mobile species within very large marine protected areas, data from array‐based acoustic telemetry can be analyzed using dynamic Brownian bridge movement models, which account for the distance and elapsed time between consecutive detections and can establish the extent of an animal's home range that is encapsulated within the protected area (e.g., Carlisle et al., 2019), although note earlier discussion around the challenges in doing this. For the shark species within this study, it was estimated that gray reef sharks *Carcharhinus amblyrhynchos* (Bleeker 1856) required at least 1 year, and silvertip sharks *Carcharhinus albimarginatus* (Rüppell 1837) 2 years of monitoring to effectively estimate their activity spaces (Carlisle et al., 2019). Alternatively, even species capable of making long‐distance movements well beyond the range of acoustic receivers may show high levels of residency or site fidelity to specific places and at specific times of year (Curnick et al., 2020), which may be sufficient to offer a degree of protection during important behaviors or key life‐history stages. Thus, assessing the space use of multiple species concurrently can help to demonstrate the enhanced efficacy of marine spatial protection, particularly as marine protected areas are rarely established with a single species in mind (Casselberry et al., 2020; Hays et al., 2020). Once a tagged fish moves outside of the range of a receiver, however, there is a significant degree of uncertainty; even notoriously site‐faithful *C. amblyrhynchos*, for example, can appear to undertake different scales of “long‐range” movements (134 km derived from acoustic telemetry [Heupel et al., 2010] and 926 km derived from satellite tracking [White et al., 2017]). This is beginning to be remedied, in part, through cross‐boundary tracking initiatives such as the FACT Network, the Integrated Tracking of Aquatic Animals in the Gulf of Mexico (iTAG), OTN and the Integrated Marine Observing System (IMOS), but remains an issue for non‐networked, isolated, or remote protected areas. AT remains a powerful and persuasive tool for quantifying full or partial space use inside current or proposed protected areas (Barnett et al., 2012; Knip et al., 2012), movements between different management zones operating as a network (Espinoza, Lédée, et al., 2015), estimation of species‐specific risk from illegal fishing activity (Jacoby et al., 2020), and improving spatial conservation by directly informing policy (Lea et al., 2016).

##### Human–wildlife conflict

Establishing the cause and effect of human–wildlife conflict in aquatic environments remains challenging and is infrequently documented. Additionally, the (often) passive nature of more recent AT studies means that data are rarely available to inform real‐time responses to potential conflict. However, the network of arrays around the coast of Australia that comprise the IMOS (formerly the Australian Animal Tagging and Monitoring System) offer an exception to this general trend. Over the last decade, passive arrays in Western Australia have been supplemented with satellite‐linked Innovasea VR4 Global receivers at some of the most popular beaches for people (McAuley et al., 2016). Providing near real‐time data retrieval, AT is being linked to social media platforms to generate “live alerts” to beach goers when white sharks *Carcharodon carcharias* L. tagged with acoustic transmitters approach the area. Building on the back of a large collaborative research programme, the Shark Monitoring Network initiative has informed thousands of water users about hundreds of potential “shark hazard events” (McAuley et al., 2016). The advent of increasingly accessible, real‐time data, however, is not without its potential problems, with these same data being used to locate and kill “problem individual” sharks, undermining not only the safeguarding intentions of the initiative, but also the science and the conservation behind the project (Meeuwig et al., 2015). This has led to calls for a more proactive approach to mitigating the potential unintended consequences of animal tracking and the associated data use, which may manifest as increased exploitation and disturbance of threatened species (Cooke et al., 2017).

Elsewhere, within recreational catch‐and‐release fisheries, estimates of post‐release survival are often inaccurate, with mortality sometimes occurring immediately, for example as a result of barotrauma, or a short while after as stress and injury from capture make individuals more susceptible to depredation (Raby et al., 2014). Quantifying the extent and timescale of mortality, however, remains a challenge but fortunately one where AT is beginning to make inroads. It was recently estimated, using a 3D acoustic positioning array in the Gulf of Mexico, that 83% of red snapper *Lutjanus campechanus* (Poey 1860) and 100% of gray triggerfish *Balistes capriscus* (Gmelin 1789) mortality was a result of post‐release depredation. However, for snapper at least, releasing individuals with descender devices (weighted devices that assist in returning the fish to depth) did significantly reduce mortality (Bohaboy et al., 2020). It is important to remember, of course, that once collected, AT data might also reveal unintended insight. The near‐simultaneous loss in December 2014 of 15 acoustic transmitters from an array in a protected area in the central Indian Ocean, for example, was found to be indicative of a suspected illegal fishing event once natural tag loss from the system had been controlled for (Tickler et al., 2019). As pressure on aquatic resources continues to increase, as well as increasing potential for distributional shifts of species in response to climate change, we envision that issues around human–wildlife conflict will continue to increase, presenting further opportunities for AT to play a role in monitoring and mitigation.

#### 1.2.2 Kinematics, energetics, and physiological impacts of human‐modified systems

In its simplest form, AT enables an individual to be detected at two spatially and temporally separated points, allowing estimation of minimum distance moved over time, that is, swim speed over ground, and thus broad inference about behavioral state and energy costs in free‐swimming fish (e.g., Madison et al., 1972). The more spatially and/or temporally separated these detection events are, the larger the error in such estimates due to failure to capture variations in path curvature and depth, as well as behaviors such as resting and burst swimming (Cooke, Thorstad, & Hinch, 2004). The increasing resolution and near‐continuous positioning afforded by dense passive receiver arrays and active tracking technologies enable more accurate determination of swim path metrics such as speed, turn angle, and direction of movement, although active tracking can practically only achieve this for a small number of individuals over limited temporal and spatial scales (Meese & Lowe, 2020). From these, key descriptors of path characteristics (e.g., tortuosity) can be derived to determine how well a track conforms to established movement models (e.g., correlated random walk, biased correlated random walk, Lévy walk), helping to develop more accurate models of dispersal (Papastamatiou et al., 2011).

Overlaying fine‐scale (±<5 m) 2D and 3D individual trajectories from acoustic positioning with concomitant environmental data has proven key to understanding the mechanisms underpinning individual behavioral responses to anthropogenic perturbations. For example, near‐continuous tracks of migratory European eel *Anguilla anguilla* and *S. salar* have been analyzed in relation to flow fields on their approach to hydropower and water‐withdrawal facilities. These study systems have proven significant in unraveling the complex interactions between fish and the multiple hydrodynamic variables that elicit behaviors such as rejection on the approach to accelerating flows (Piper et al., 2015), milling (Svendsen et al., 2011), and fine‐scale adjustments in swimming direction and speed (Silva et al., 2020). Furthermore, precise, real‐world data are invaluable for the parametrisation and validation of agent‐based models. Predictive behavioral models that enable testing of different management scenarios aimed to reduce fish mortality and delay are a key area of focus for hydropower, water abstraction, and flood defense managers (Goodwin et al., 2006, Andrew Goodwin et al., 2014).

Even at fine resolution, however, inferences about the energetics of movements and behaviors derived from position data alone will be inherently lacking through failure to consider the dynamics of the fluid in which the fish is moving and the physiological state of the individual. Thorough understanding of the biomechanics and energetics of free‐swimming fish therefore requires moving beyond an animal's track characteristics. Measurement and modeling of salient metrics of the surrounding hydrodynamic environment such as flow velocity, turbulence intensity, and hydraulic strain have revealed much about how migrating fish attempt to optimize energy usage (Piper et al., 2015; Silva et al., 2020; Svendsen et al., 2011). For example, the modeled energy costs of a pallid sturgeon *Scaphirhynchus albus* (Forbes & Richardson 1905) actively tracked during its upstream spawning migration through a velocity‐surveyed section of the Missouri River, United States, were lower than those calculated for 10^5^ random paths in the same reach (McElroy et al., 2012). A suite of fish‐borne sensors enabled time‐stamped monitoring of an individual's physiological processes such as muscle activity (Cooke, Thorstad, & Hinch, 2004), heart rate (Lucas et al., 1991), and tail‐beat frequency (Watanabe et al., 2012), while accelerometers and speedometers provide a measure of speed (Block et al., 1992). These have been used successfully alongside acoustic positioning techniques to explore fish activity patterns and their associated energy expenditures (Meese & Lowe, 2020), as well as the stress responses and energy costs resulting from human disturbances such as recreational fishing (McLean et al., 2019), hydropower generation (Burnett et al., 2014), and seismic surveying (Davidsen et al., 2019). While such technologies began as stand‐alone and typically data‐storage devices (Cooke, Thorstad, & Hinch, 2004), the evolution of transmitting sensors and those integrated within acoustic positioning technologies offer much greater scope to derive detailed data from free‐swimming fish without the need for recapture (Cooke et al., 2016; Lennox et al., 2017). Furthermore, rapidly evolving data compression and transfer techniques to embed additional sensor data within the transmitted acoustic signals will serve to deepen our mechanistic understanding of fishes' behaviors as they move through their increasingly human‐impacted environments (Steven et al., 2022).

### 1.3 Future directions and considerations

In this section we look ahead to some of the innovations that we envisage will further enhance the application of AT in fish biology. We highlight areas in which innovations are likely to have the biggest impact and discuss some of the more generic issues and considerations that still present a challenge for AT.

#### 1.3.1 Tracking small species and life stages

Historically, the large size of transmitters has biased the application of AT towards adult life stages and/or juveniles of large taxa only. Furthermore, for species that exhibit sexual body size dimorphism, such as anguillid eels, acoustic tracking has been skewed towards larger females (Bultel et al., 2014; Piper et al., 2013). This challenges the principal assumption that studied individuals are representative of the wider population and risks the erroneous extrapolation of findings. In applied research, this can have serious negative consequences, such as misdirection of conservation funds or ineffective mitigation measures. To remedy this, continuing efforts towards transmitter miniaturization, aided by substantial improvements in battery and microprocessor technologies, are greatly increasing the range of life stages and species that can be tracked (Figure 3) (Lennox et al., 2017). When studying small species and life stages for which commercially available transmitters may approach the limits of the acceptable tag to body weight ratio (traditionally the 2% rule, [Winter, 1983], although this is increasingly being questioned, [e.g., Brown et al., 1999]), body morphology also becomes an important consideration. The narrower body cavity relative to fish size among species with an elongated shape requires even smaller transmitters. New transmitters as small as 12.0 × 2.0 mm, weighing as little as 0.08 g in air and lasting 30 days at a 5‐s ping rate interval, have been recently tested in juvenile lamprey *Entosphenus tridentatus* (Richardson 1836) and *A. rostrata* (Mueller et al., 2019; Figure 3a). Although AT has been used across a wide range of taxa, the scale of investment directed towards juvenile salmonid research to assess stocks (see *Fisheries management*) and quantify anthropogenic impacts such as hydropower facilities continues to drive much of the innovation within the field (Cooke et al., 2013; Walker et al., 2016). For example, injectable acoustic transmitters have been developed for small fish sizes, but also to cope with the volume of individuals and speed required to tag statistically meaningful samples, given the high mortality rate of juvenile out‐migrating salmon smolts (Deng et al., 2015).

Long battery lives are required to track species across multiple life‐history stages. The lifetime of an acoustic transmitter, however, reflects the trade‐off between battery power and the frequency and strength of transmissions, along with any additional power burden from integrated sensors. For smaller species and life stages, the need for miniaturization inevitably results in a transmitter with a shorter battery life and typically smaller detection range. Currently, the smallest available acoustic transmitters are best suited to capturing brief windows of activity rather than providing near whole lifecycle data. Life‐time tracking will significantly improve our understanding of small and cryptic species conservation, however, and small battery‐less tag technologies, for example passive integrated transponders, remain viable on a multidecadal scale, enabling near whole lifetime studies. Near whole lifetime AT studies of small individuals may be possible in the future using self‐powered transmitters that incorporate a transducer to use the energy from fish locomotion to power the tag (Li et al., 2016). More sophisticated programming regimes, such as multiple time‐limited transmission rates and dormancy, offer researchers increasing flexibility to extend the life of small transmitters to capture discrete periods of interest. These are, at present, pre‐programmed and so require detailed a priori knowledge of predictable behaviors and/or life histories to be of most use (Davies et al., 2020; Stevenson et al., 2019). Further development of responsive acoustic transmitters that can dynamically adapt settings, for example transmission frequency or dormancy, in response to distinct changes in activity or environmental conditions such as the transition between fresh and saltwater, as has been trialed in combined acoustic and radio transmitter tags (Deary et al., 1998), would vastly improve their usefulness.

Notwithstanding the restrictions posed by transmitter size, our application of AT to small species and/or life stages is often limited by their inherent spatial ecology. The microscale movements relevant to many small fish species, for example, anemonefish *Amphiprion* sp., whose home range is often less than 1 m (Kobayashi & Hattori, 2006), are smaller than can be effectively studied given the current accuracy of most technologies. Advances in hyperbolic positioning systems have enabled researchers to reliably achieve 2D and 3D positions at submetre accuracy and precision in small individuals (e.g., Leclercq et al., 2018) (Figure 2). In a novel study, the Juvenile Salmon Acoustic Telemetry system (Lotek Wireless) was employed in a challenging open marine environment to simultaneously track large numbers of individuals as small as 90 mm (Aspillaga et al., 2021). Challenges remain for many applications, however, especially in complex habitats such as rocky areas, coral reefs, and macrophyte beds, where detections are impeded (Baktoft et al., 2015).

#### 1.3.2 Multisensor transmitters, combined technologies, and surrogates

Multisensor acoustic transmitters and AT studies that integrate additional biologging technologies (accelerometers, magnetometers, physiological sensors etc.) and in some instances direct observations, clearly facilitate broader research questions (Figure 3). This has promoted greater exploration, for example of the proximate mechanisms underpinning specific population level processes such as group living, social behavior, or individual behavioral variation/consistency through time (Villegas‐Ríos et al., 2017; Wilson et al., 2015). Knowledge of these mechanisms for specific fish populations has the potential to greatly advance how we conserve and manage commercially important or highly threatened species (Villegas‐Ríos et al., 2022). Importantly, the four major AT manufacturers (Thelma Biotel, Lotek, Innovasea, and Sonotronics) offer different sensor combinations, with some facilitating bespoke sensor integration into transmitters. Careful consideration of the end user of AT data and anticipated collaborations with other research groups is needed prior to deciding on where to source equipment. Currently, not all suppliers provide integration of all sensor combinations into their transmitters and restrictions remain around the compatibility between transmitters and receivers from different suppliers.

The recent modification and miniaturization of RAFOS technology (a form of sound fixing and ranging, SOFAR) has presented the potential to track relatively small marine fish species across large areas of the ocean. The RAFOS Ocean Acoustic Monitoring approach uses moored acoustic transmitting units emitting acoustic signals that carry up to 1000 km, offering the potential to conduct whole ocean scale tracking studies. Individual study fish are equipped with a RAFOS float receiver that detects the sound pulses from fixed stations and triangulates position. This logged information is either recovered by recapturing fish returning to known areas, for example, salmonid spawning rivers (which permits a significantly smaller tag than PSAT technologies), or can be transmitted to land via satellite after the float pops‐up at a predefined time for species able to accommodate the larger tag this requires (Bronger & Sheehan, 2019). Clearly, these innovations have the potential to provide much greater insight into highly migratory species, particularly those that face multiple threats during long‐distance movements.

Despite many encouraging examples within the literature where technological innovation or integration of sensors has provided true insight and/or policy‐relevant data, combining technologies may not be a viable solution in instances where mortality is high (Klinard & Matley, 2020). Ethical, logistical, and financial drivers are increasingly promoting approaches that reduce, or even remove, the requirement to capture and tag live fish to derive biologically meaningful data. For example, in perilous scenarios such as during transit of water control and power‐generation infrastructure, multisensor passively conveyed devices have been employed to collect environmental data on the likely experience and fate of fish (Deng et al., 2017; Pflugrath et al., 2019). By incorporating key locomotory and behavioral characteristics, it is hoped that evolving robotic fish surrogates (Figure 3c), combined with computational fluid mechanics and predictive modeling, will ultimately eliminate the need for live fish transit experiments at hydropower facilities (the RETERO project, https://retero.org/). Many of the research areas discussed may be advanced by applying increasingly sophisticated analyses to historic acoustic telemetry datasets and by combining biological, physiological, and behavioral data to produce predictive models to allow scenario testing of management interventions, thus greatly reducing the costs and animal use associated with the traditional “build and test” approach (Andrew Goodwin et al., 2014; Snyder et al., 2019).

#### 1.3.3 Live data for near real‐time management

AT systems which instantaneously relay detection data to a computer or data transfer unit at the surface present an opportunity for assessment of and dynamic adaptation to activities that may be stressful, harmful, or fatal to fish. So‐called “live” AT technologies mean fish tracks can be reconstructed, in near real time, to measure the impact on fish of human disturbance activities such as marine infrastructure development (e.g., pile driving, gas and oil exploration and extraction, wind farms, and port development). The potential for this approach is in its infancy, but has been installed as part of the innovative adaptive planning consent process for a major road/airport infrastructure scheme with potential to disrupt important salmonid migration routes in a Norwegian fiord (Davidsen et al., 2021). Data retrieval, however, continues to be a limiting factor for many AT studies that would benefit from live or near‐live upload. In many instances, data retrieval can be extremely expensive and/or unreliable. Consequently, there has been significant interest in innovation that can provide reliable, real‐time, long‐range wireless access to AT systems. A recent proof of concept of the internet of fish uses low‐power wide‐area networks and long‐range wireless data protocols with low‐power modulation to achieve just this, presenting an exciting opportunity for long‐term, real‐time behavioral monitoring of fish in commercial settings, for example (Hassan et al., 2019). The implications of this innovation could be huge for improving fish welfare in intensive aquaculture. With increased global scrutiny around the ethics of intensive fish farming it seems likely that AT technologies could become a routine tool to manage and demonstrate fish welfare (Matley et al., 2021).

#### 1.3.4 Accuracy, precision, and validation

Irrespective of the scale and complexity of a receiver array or the study question being addressed, robust interpretation of animal movement data requires some quantitative measure of the accuracy and precision at which a transmitter can be detected. Crucially, this should capture the influence of spatial and temporal variation on detections within the specific study environment. Such sources of detection error are frequently overlooked or only partially accounted for in acoustic tracking studies (Brownscombe, Griffin, et al., 2019; Kessel et al., 2014; Klinard et al., 2019). Equally, reflecting on detection efficiency during a study might also reveal redundancy within the array design (Gabriel et al., 2021) that, once identified, might free up a proportion of valuable receivers to monitor new locations.

Advances in transmitter and receiver design and data processing techniques provide increasing capability to achieve high accuracy and precision from both cabled and non‐cabled arrays. For example, more sophisticated transmitter programming has reduced data loss from transmission collision when multiple transmitters are present and increased detection probability and positioning accuracy (Cooke et al., 2005), even in acoustically noisy environments (Bergé et al., 2012; Leander et al., 2019; Weiland et al., 2011). Fine‐scale positioning studies typically require substantial post‐processing to derive 2D or 3D positions from detection data, but the continual refinement of positioning methods is improving accuracy and reducing data omission during this process. For example, by employing a time‐of‐arrival rather than time‐difference‐of‐arrival algorithm and incorporating a random walk movement model, the yet another positioning solver approach developed by Baktoft et al. (2017) outperformed comparable methods in terms of both accuracy and the number of positions resolved, a method that has been successfully applied to acoustically reflective environments (Vergeynst et al., 2020). On a broader scale, where receivers may be dispersed over a wide area, model simulations that predict each receiver's theoretical detection range based on site‐specific architecture, environmental variables, and target species characteristics can be useful at the design stage (Gjelland & Hedger, 2013; Hobday & Pincock, 2011). Subsequent parametrization with empirical environmental datasets and detection range tests collected within the study enables calibration of live animal detection data post‐collection. Brownscombe, Griffin, et al. (2019) developed an approach that uses variation in the detection efficiency of fixed‐reference transmitters collected at a subset of representative “sentinel receivers” as a proxy measure for detection range across the whole array. Application of the detection range correction factors they generated to a data set on *T. falcatus* from the Florida Keys showed substantial departure from the raw data (up to 127%), with most difference in the space use patterns associated with habitat and diel differences (Brownscombe, Griffin, et al., 2019).

## 2 CONCLUSIONS

Meeting the needs required of our rapidly changing aquatic environments, and doing so in ways that are fair, equitable, sustainable, and responsive, is not trivial. In 2017, Lennox et al. (2017) set out a vision for how multiplatform tracking systems will be utilized in the future to simultaneously monitor the position, physiology, and activity of aquatic animals and their environments. They highlighted the four pillars of progress required to achieve this: “(1) technological and infrastructural innovations; (2) transdisciplinary integration of collected data and new methods of analysis; (3) emergent applications for telemetry data in fisheries, ecosystems, and the global management of aquatic animals; and (4) looking forward to solving challenges that currently inhibit progress in telemetry research” (Lennox et al., 2017). Since then, there have been advances in AT technology, data integration, analyses, and application, many of which we have tried to cover in this review, but all of which have significantly progressed research within the key themes discussed (see summary in Table 1).

As AT users continue to diversify, alongside an ever‐growing list of analyses and packages designed to handle the associated data, there is a need to consolidate the current state of the field of AT, which remains a “go‐to” approach for addressing key questions within fish biology and conservation. This comes at a time when the pathway from fundamental species ecology to end‐user management and policy making is clearer than ever before; careful consideration of AT application, study design, and interpretation, including the potential pitfalls, is needed to ensure transparency during all stages of this process (Brownscombe, Lédée, et al., 2019). As we outline here, AT is both broadly applicable and highly nuanced, enabling us to tease apart patterns of space use, segregation, and migration, and through increasingly more accurate high‐resolution tracking, interactions and associations between individual fish. Combined with machine learning approaches, physiological or energetic sensors, or by coupling with ecotoxicology, eDNA, or stable isotope analyses, AT can be an even more powerful approach for monitoring the behavior of individuals and groups of fish. As both technological and analytical developments continue apace, this is an exciting time to track fish using acoustics. We hope that the field will continue to attract innovation that will generate new insight for mitigating threats, managing our stocks, and protecting the species occupying imperiled aquatic environments.

## AUTHOR CONTRIBUTIONS

DMPJ conceived and structured the review. Both DMPJ and ATP contributed equally to the writing and editing of the manuscript.

## Data Availability

No data were used in this review paper, which discusses work that is already published.
